# Visualizing active fungicide formulation mobility in tomato leaves with desorption electrospray ionisation mass spectrometry imaging†

**DOI:** 10.1039/d4an01309c

**Published:** 2024-11-18

**Authors:** Akhila Ajith, Emrys Jones, Emily Prince, Drupad K. Trivedi, Giles N. Johnson, Phillip J. Milnes, Nicholas P. Lockyer

**Affiliations:** a Photon Science Institute, Department of Chemistry, University of Manchester Manchester UK nick.lockyer@manchester.ac.uk; b Waters Corporation Altrincham Road Wilmslow UK; c Syngenta, Jealott's Hill International Research Centre Bracknell UK; d Manchester Institute of Biotechnology, Department of Chemistry, University of Manchester Manchester UK; e Department of Earth and Environmental Sciences, University of Manchester Manchester UK

## Abstract

Newer and safer agrochemicals are always in demand to meet the increasing needs of a growing population for affordable food. Spatial chemical monitoring of the active mobility of an agrochemical is essential to this agrochemical development process and mass spectrometry imaging (MSI) is proposed as a safer, easier alternative to the existing standard of autoradiography for the same. With desorption electrospray ionisation mass spectrometry imaging (DESI MSI) using leaf imprints, we were able to visualize the active agrochemical mobility of a commercial fungicide formulation with the active ingredient Azoxystrobin in whole tomato leaves. The leaf-imprinting method was optimized with precise control over the pressure conditions and time of imprinting to yield highly consistent samples for imaging. The reproducibility of this method was tested with the Azoxystrobin formulation applied to tomato leaves and was compared to the mobility of the unformulated Azoxystrobin standard in similar application conditions. The xylem mobility and the lateral-leaf lamina spreading of the fungicide were visualized with mass spectrometry imaging and validated using complementary LC-MS studies. The necessity and importance of the agrochemical application as a formulation were re-iterated by the limited mobility observed in Azoxystrobin standard studies compared to the Azoxystrobin formulation. This mass spectrometry imprint-imaging method could be translated for the visualization of any xenobiotic in further foliar systems particularly with soft leaves.

## Abstract 2

Mass spectrometry imaging can be used to reveal the active mobility of an applied agrochemical and co-formulants in a plant.

## Graphical abstract

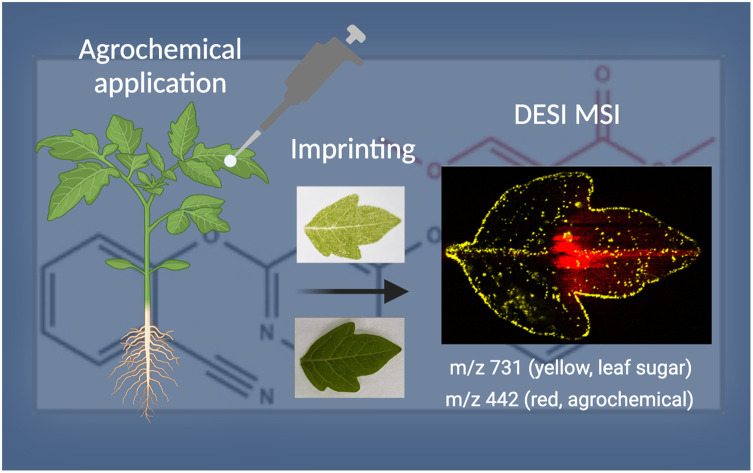

## Introduction

The search for an ideal *in planta* imaging method for agrochemicals has been extensive owing to the great advantages it might offer in understanding the spatiotemporal mobility of applied agrochemicals in plants.^1^ Agrochemicals when applied to the plant, contain one or more active ingredients (AI) responsible for the principal agrochemical activity, usually along with several other co-formulants which might help in improving the effectiveness of the AI(s) including surfactants and oils.^2^ Monitoring the active mobility of such agrochemicals in a plant with sensitivity, specificity and high resolution in real-time is a challenging but necessary task. Previously, autoradiography has been the go-to method to study agrochemical mobility in leaves.^3^ However, this is a time-consuming and laborious process that requires the synthesis of the radiolabelled equivalent of the chemical being tested and as such is usually limited to the far end of product development. Mass spectrometry imaging (MSI) is a well-established label-free spatial chemical mapping technique used for many kinds of studies including disease progression,^4,5^ endogenous metabolites imaging,^6^ drug distribution analysis,^7^ agrochemical distribution testing,^8^ semiconductor analysis,^9^ archaeological sample imaging,^10^ food testing^11^ and can be even used as an intraoperative diagnostic tool.^12^ There are various kinds of MSI techniques available in addition to DESI MSI, including matrix-assisted laser desorption ionisation (MALDI)^13^ and secondary ionisation mass spectrometry (SIMS).^14,15^ MSI as an analytical method to map agrochemical mobility and understand bio-kinetics has enormous potential in all stages of the research and development of agrochemicals due to its relatively fast and high-throughput experimental methods compared to autoradiography. The sample preparation and analysis protocols for analysing animal and human tissue samples are quite well established often involving cryo-sectioning, thaw-mounting and desiccation, followed by analysis with the appropriate MSI method, with or without matrix spraying.^16^ However, the use of MSI to understand the agrochemical mobility in plant leaves has been limited due to the lack of standard sample preparation protocols and analysis methods which are quite distinct from that of the mainstream animal tissue MSI. This is mainly due to the delicate structure of the leaf which makes it difficult to cryo-section. The uneven waxy surface of the leaf poses a challenge to studying the sub-surface absorption and mobility of applied agrochemicals in the lamina of the leaf through the classical sample preparation strategies.

Compared to animal and human tissue, there are relatively few MSI studies reporting on agrochemical analysis on plant leaves. Among the few published works, the earliest study to our knowledge was done using soy plants looking at the mobility of Mesotrione and Azoxystrobin with direct and indirect MALDI MSI (leaf blot analysis).^17^ Most MSI studies on foliar agrochemical mobility have been done with MALDI, presumably due to the prevalence of MALDI as a favoured MSI tool widely.^14^ However, the interference of matrix peaks in the low mass range and the relatively more complex sample preparation makes DESI an attractive alternative for MSI of low mass analytes. Direct analysis of leaf surfaces is futile to understand the active agrochemical mobility of systemic agrochemicals as most of the active mobility happens sub-cuticle. Although there have been efforts to overcome the cuticular barrier to probe the true active distributions in leaf bulk with chemical^18^ or physical removal^19^ of the waxy cuticle, the chances of introducing sample preparation artefacts are quite significant. Indirect MSI with imprints of leaves on appropriate surfaces can be an alternative method for looking at sub-cuticular distributions for softer, fleshier leaves as it reduces the 3-D distribution of the leaf onto an easily analysable 2-D surface such as porous PTFE sheets.^20,21^ However, the choice of imprinting surface, the method of imprinting, along with the porosity of the surface taken and the pore volume may affect the quality of the imprint. Improper choice of imprinting surface, pressure and time of imprinting may result in inefficient imprints, which might not reflect the true 3-D distribution in the leaf and may cause spilling of leaf sap and the loss of spatial information.

In a study which performed a direct comparison of DESI, autoradiography and PET by Jacobsen *et al.*, it was shown that similar or more reliable chemical information can be obtained with DESI with respect to autoradiography at a fraction of the cost although the sensitivity of DESI might be lower than autoradiography.^22^ With the current developments in DESI MSI pushing the spatial resolution limit to tens of microns,^23^ DESI MSI has the ability to image at much better spatial resolutions than obtainable with traditional autoradiography. Along with the time, cost of operation of a radiochemical handling facility^24^ and toxicity involved, autoradiography is also restricted by chemical specificity^25^ and the number of components detected in an agrochemical formulation as usually, only the AI is radiolabelled, and its radioactivity is followed by autoradiography in one single experiment. Any metabolites which do not retain the radiolabel will not be detected. Due to the amount of resources required to do an autoradiography study, such studies are restricted to the far end of product development with targeted aims, and are not usually done for an exploratory study. With minimal technical requirements, a DESI MSI experiment requires a fraction of the cost and can be an attractive alternative to autoradiography, making spatial mobility studies possible at all stages of product development. With the usage of the same PTFE substrate for the imprinting of leaves, the need for sample dependent optimization of DESI spray conditions and geometrical parameters are also minimized for an easy and high throughput spatial imaging.

**Fig. 1:**
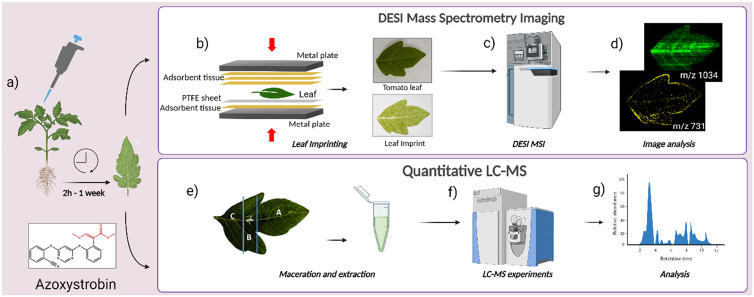
Workflow of experiments in this study. (a) AMISTAR formulation/Azoxystrobin standard application on leaf and sampling at 2 h to 1 week after application. (b) Imprinting of young tomato leaves to obtain imprints of the leaf. (c) DESI MSI imaging of the leaf imprint. (d) Extraction and analysis of DESI MSI images from leaf imprints. (e) For LC-MS study, the leaf was divided into three parts, A – the area towards the periphery of the leaf, B – the area in and around the point of application of the formulation and C – the area towards the stem of the leaf. The leaf sections were then macerated and leaf extracts were used for LC-MS experiments. (f) Quantitative LC-MS experiments with leaf extracts of different parts of leaf at various time points. (g) Analysis of LC-MS data. This image was created using BioRender.com.

In this work, we have looked at a widely studied^26,27^ strobilurin fungicide^28^ Azoxystrobin (Fig. 1a) and co-formulants (Table S1†) in a commercial fungicide formulation to demonstrate a highly reproducible sample preparation and user-friendly methodology to study systemic agrochemical mobility in young tomato leaves using leaf imprints and DESI MSI. Azoxystrobin acts as a fungicide by binding to the Q_o_-site of the complex bc_1_ hindering the electron transport between cytochrome b and cytochrome c in the mitochondrial respiratory chain.^29,30^ The results of the DESI MSI study were validated by the well-established LC-MS method. Hence, by testing pressure and time conditions for imprinting and with careful choice of a microporous PTFE sheet as an imprinting surface, it was possible to visualize the active spatiotemporal mobility of a systemic agrochemical formulation through entire leaves for the first time.

## Materials and methods

In this study, we used the DESI MSI technique to image the foliar mobility of the active ingredient, Azoxystrobin (Fig. 1a) in a commercial fungicide formulation, AMISTAR manufactured by Syngenta Ltd in young tomato leaves. The experimental and instrumental conditions were optimised to yield maximum sensitivity for Azoxystrobin including the solvent system for DESI and the geometrical parameters for DESI source set-up.

### Chemicals and reagents

Collaborators at Syngenta Ltd provided AMISTAR formulation and Azoxystrobin standard was obtained from Sigma-Aldrich, UK. All other chemicals and UHPLC or HPLC grade solvents were obtained from commercial sources unless stated otherwise. The Polyflon FLONTEX™ 100-5 PTFE sheets were obtained from Polyflon Technologies Ltd, Stafford, UK.

### Plant leaf samples

The study used young tomato plants in their two-leaf stage. The tomato plant was selected for this study as it is a common dicot plant cultivated widely. For the experiments, tomato plants were grown with commercially available tomato seeds (Gardener's Delight, Johnsons seeds, UK) on Levington's multi-purpose compost. During the growing period, the temperature conditions of 18/22 °C for night/day were maintained with the light intensity of 100 μmol m^−2^ s^−1^ over the day length of 16 hours.

### Fungicide application

AMISTAR is a broad-spectrum fungicide formulation used to treat a range of fungal diseases. It is commercially available as a suspension concentrate in the composition of 250 g L^−1^ of Azoxystrobin. For application in young tomato leaves, a solution of 2500 ppm Azoxystrobin containing formulation in water was taken. All tomato plants used in this study were taken at 17 days after planting of the seeds when typically the first pair of true leaves are well-developed and a single droplet of 10 μL, 2500 ppm formulated Azoxystrobin in water was applied near the centre of one leaf on or/and near the mid-vein. The leaves were left undisturbed until sampled after 2 h, 24 h, 56 h and 1 week after formulation application. The Azoxystrobin formulation droplet was visible on the leaf after drying. A similar application procedure was also followed for the Azoxystrobin standard study wherein, 10 μL, 2500 ppm Azoxystrobin standard solution in water was applied as a single droplet to one leaf on the 17^th^ day after planting of the seeds.

### Imprinting of leaves

On reaching a sampling time point, the plant with the leaf to be sampled was uprooted and immediately transported from the plant room to the sample preparation area in the analysis lab in a box maintaining pristine conditions. The microporous PTFE sheet to be imprinted on was cut into an appropriate-sized piece depending on the size of the leaf to be imprinted. The Polyflon FLONTEX™ 100-5 PTFE sheets used in this study has a functional pore size of 1 to 2 μm, a pore volume of 40% and a thickness of 0.38 ± 0.05 mm. The imprinting was done using a commercial heat press machine, (7 Ton 600W Rosin Extractor with Dual 2.4 × 4.7 Inch Heating Plates, Shenzhen LTQ Vapor Electronics Co., Ltd, China). This heat press offered precise control over the pressure, temperature, and time for the imprinting of leaves and was found to be more effective and reproducible than a table vice commonly used for leaf imprinting MSI studies.^21^ The imprinting process was performed by keeping the adaxial side of the leaf (Fig. S1†) towards the PTFE sheet and then sandwiching it between layers of adsorbent tissue paper (Kimtech delicate task wipes) to collect any excess liquid coming out from the leaf during the imprinting process (Fig. 1b). This was done to mitigate the risk of compound delocalization due to liquid exudation. For soft tomato leaves in the two-leaf stage, a pressure of 2000 psi applied uniformly for 1 minute at ambient temperature produced a good imprint with visual similarity in morphology to the leaf, which was usually replicated even in the ion images produced from the imprint (Fig. 1b–d). The optimisation of the imprinting method was done based on previously reported indirect DESI MSI studies with leaves.^31^ Several time periods of imprinting from 30 s to 2 min and, pressures from 500 psi to 2500 psi pressures were tested for the leaves in the growth stage described in this study. From the different combinations of time and pressure conditions tested, 1 minute with 2000 psi pressure gave consistently good imprints, characterized by the visual similarity of the imprint to the leaf imprinted as shown in Fig. 1d with no leaf sap spilling observed outside the boundary of the leaf. The slight variability in leaf cuticle thickness and sizes of leaves may cause slight variability in imprinting efficiency and can be a source of potential variability. Since we observed clear peaks with reasonable intensities from the tomato leaf imprints created in room temperature conditions, application of heat during the imprinting process was not further explored. However, the application of heat might be beneficial to improve the imprinting process for dryer leaves with thicker cuticles. The leaf imprints were left for at least 10 minutes to air-dry before being analysed with DESI MSI.^31^ For analysis, the leaf imprints were either fixed using a double-sided carbon tape (Agar Scientific Ltd, Essex, UK) to a standard microscope slide (76 mm × 25 mm) or a full-stage DESI slide provided by Waters Corp. (analysable area of 76 mm × 61 mm) depending on the size of the leaf taken for the experiment. All leaf imprints used in this study fit into either the standard microscope slide or the full-stage DESI slide. If the leaf dimensions exceed the largest analysable area possible in the DESI stage, the imprint of the leaf created with an intact, full leaf can be dried and cut into smaller analysable areas for multiple imaging experiments. However, this needs to be tested further as it was not relevant in the study described here. All DESI experiments were done within 24 hours of imprinting, although significant changes to signal localization or image quality were not observed even when analysing the imprints after 1 week of imprinting (Fig. S2†). Re-analysis of leaf imprints with DESI also yielded meaningful images comparable to the initial analysis indicating that the imprints of the leaf could be stored for at least for a week in ambient conditions and re-analysed. Further studies need to be conducted to test the shelf life and viability of leaf imprints over extended periods.

### DESI MSI of leaf imprints

The imprint imaging was performed using a modified commercial DESI source (Prosolia Inc., Indianapolis, IN, USA) with a heated inlet fitted with a high-performance emitter cartridge (Waters Corporation, Milford, Massachusetts, USA) coupled to a high-resolution time-of-flight mass spectrometer (Synapt G2-Si mass spectrometer, Waters Corporation, Milford, Massachusetts, USA). The imprints were scanned in positive ion mode in 2-D over the *m*/*z* range of 0 to 1200. The ion images were acquired with a pixel size and approximate spray spot diameter of 100 μm. The chemical image was acquisitioned with a speed of 1000 μm s^−1^ using 80 : 20 (v/v) methanol/water (MeOH/H_2_O) with 0.1% formic acid used as spray solvent with a flow rate of 2 μL min^−1^ assisted by a co-axial sheath gas flow of 0.25 MPa, and a spray potential of 0.5 kV. The reason for the choice of solvent is explained in ESI Note 1 and Fig. S3.† The spray solvent also contained Leucine Enkephalin (Waters Corporation, Milford, Massachusetts, USA) in a concentration of 20 pg μL^−1^ for post-acquisition lock mass calculations. The inlet capillary taking secondary droplets to the MS inlet was heated to 400 °C to aid desolvation with the MS inlet maintained at 150 °C. The geometrical parameters of the acquisition were kept the same across the different imaging experiments which include, the angle of spray at 65°, the spray-to-surface distance of approximately 3 mm and inlet to spray distance of approximately 5 mm. The data acquisition was done using the Waters Corp. software, the high-definition imaging (acquire option in HDI) and the MassLynx V4.1 software at a mass resolution of 15 000–20 000 (FWHM at *m*/*z* 556.2771). The acquired raw data was processed using the process option in HDI software extracting the most intense 1500 *m*/*z* values and applying lock mass conditions to adjust for any mass shifts during data acquisition. The data files were read using the analyse option on the HDI software showing the most intense 1500 ion signals and all the ion signals of interest analysed in this study were from this list for all DESI images. The HDI software also allows for region of interest (ROI) selection, which then creates a .raw file containing the average mass spectrum for the selected region openable in the MassLynx software. The imaging data files were also readable using the Bruker SciLS lab Pro software providing the capability to do ROC (receiver operating characteristics) analysis on images (Fig. S7†) and ion correlation matrix calculation comparing different ion signals (Fig. S8†).

### Quantitative LC-MS of leaf extracts

To complement the DESI MSI studies of leaf imprints, a quantitative LC-MS study was done by dividing the applied leaves into 3 parts as shown in Fig. 1e, A – the area of the leaf towards the periphery, B – the area of application of formulation and immediate neighbourhood and, C – the area of the leaf toward the stem of the plant. The leaves were divided so that we observed differential distribution of ion signals from formulated Azoxystrobin in these three regions. The same fungicide application method as done for the DESI MSI experiments for the leaf imprints was used to prepare the samples for the LC-MS study. However, instead of imprinting, the leaves were sectioned and stored in 2 mL extraction tubes (FastPrep® 2 mL, MP Biomedicals, Irvine, CA, USA) containing ¼ inch ceramic bead at −80 °C until taken for LC-MS study. The samples were thawed to room temperature the day before the studies and were stored at 4 °C till taken for further sample preparation. Before the LC-MS analysis, the extraction tubes with the leaves were added with 1 mL of 80 : 20 (v/v) acetonitrile/water (ACN/H_2_O) and macerated using the FastPrep 24 5G machine (MP Biomedicals, Irvine, CA, USA) with 3 × 20 seconds cycles at 4 m s^−1^. Subsequently, the extraction tubes were taken for centrifugation on a Thermo Scientific™ Pico™ 17 microcentrifuge at 10 000 rpm for 15 min. The supernatant obtained after the centrifugation step was diluted 1 in 10 with 80 : 20 (v/v) ACN/H_2_O and was transferred to HPLC vials for LC-HESI-MS analysis on the Q-exactive hybrid quadrupole-orbitrap mass spectrometer (ThermoFischer Scientific, Newington, NH, USA) attached with a Thermo Vanquish LC system having an Acquity BEH C18 1.7 μm, 50 × 2.1 mm column. The analysis was done with an injection flow rate of 0.7 ml min^−1^ and HESI conditions of sheath gas, auxiliary gas and sweep gas, flow rates of 58, 15 and 3 arb units. The auxiliary gas temperature was set at 450 °C with a capillary temperature of 320 °C and a capillary voltage of 3.5 kV. The experiment was done in an *m*/*z* range of 100 to 900 with a mass resolution of 35 000 in positive ion mode. For absolute quantification, Azoxystrobin standards in varying concentrations from 5 μg mL^−1^ to 0.00244 μg mL^−1^ were used as calibration standards to identify the amount of AI (Azoxystrobin) recovered from different parts of the leaf at various time points from the prepared samples.

## Results and discussion

**Fig. 2:**
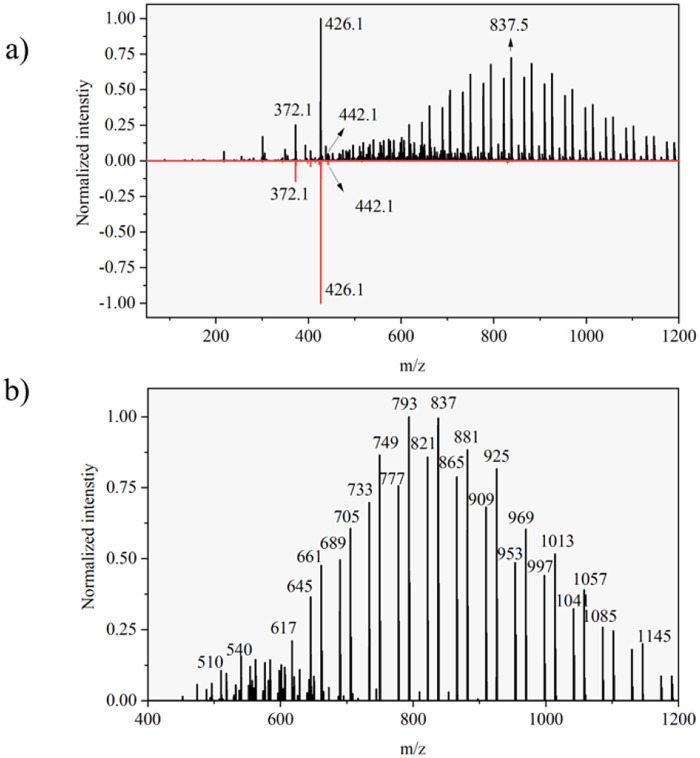
(a) A comparison of the average DESI mass spectrum of Azoxystrobin standard and the AMISTAR formulation containing Azoxystrobin acquired with the modified Prosolia DESI source on a Waters Synapt G2-Si mass spectrometer. (b) Peaks which are exclusively present in the average spectrum of AMISTAR formulation and not the Azoxystrobin standard. The inset labels of (a) and (b) correspond to the *m*/*z* value of the adjoining peak.

For reference, DESI spectra for formulated Azoxystrobin and Azoxystrobin standard compound on a glass slide were acquired and compared using the same experimental and geometrical parameters as used for DESI imaging of leaf imprints. The comparison of the DESI spectra of formulated Azoxystrobin and Azoxystrobin standard (ESI Note 2†) was used to identify formulation-exclusive ion signals (Fig. 2), the majority of which may be polymeric and may correspond to component 3 listed in the constituents of formulated Azoxystrobin (Table S1†).

### Visualizing mobility of Azoxystrobin formulation

**Fig. 3:**
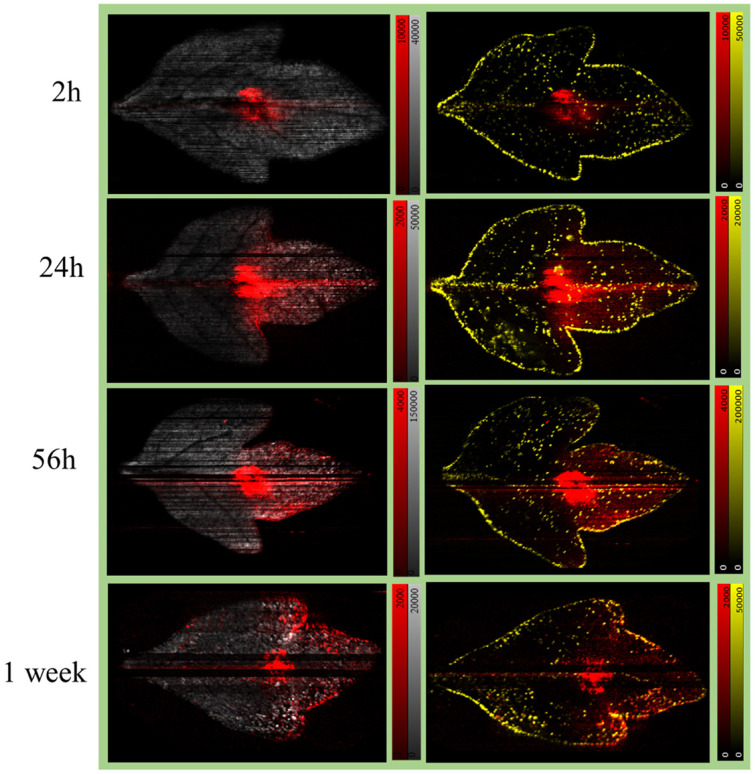
Visualizing Azoxystrobin (AZ) mobility in leaf imprints with DESI after application of formulated Azoxystrobin. A leaf from each tomato plant in the set of plants was applied with 10 μL of AMISTAR formulation containing 2500 ppm of Azoxystrobin on the 17^th^ day after planting of seeds. The formulation applied leaves from the plants were sampled and imprinted at 2 h, 24 h, 56 h and 1 week after the time of application. The obtained imprints were imaged within 24 hours post imprinting to obtain the above ion images. In the ion images, the red signal is *m*/*z* 442.1 [Azoxystrobin, potassium adduct], the yellow signal is *m*/*z* 731.3 [acyl sugar S4:21, potassium adduct] and the grey signal is *m*/*z* 1034.6 [Alpha Tomatine, proton adduct]. In the images, it could be seen that the red signal is progressing towards the periphery of the leaf as time passes with laminar spreading in the direction of movement. In the ion images, the maximum arbitrary intensity value, and the minimum arbitrary intensity value (0 in all cases) are shown with the time of sampling shown as an inset in the ion images.

Formulated Azoxystrobin applied tomato leaves were used to create whole leaf imprint images with DESI MSI as shown in Fig. 1b for various ion signals of interest. The identity of the mentioned leaf marker ions and Azoxystrobin ions was verified by MS/MS (Fig. S4†) on leaf imprints or already existing literature.^32^ The point of application of the formulated Azoxystrobin is visible in all DESI imaging experiments as an area (towards the centre of the leaf) with a high intensity of *m*/*z* 442.1 signal. Among the various adducts formed by molecular ions of Azoxystrobin, the potassium adduct, *m*/*z* 442.1 is the most intense in the DESI images, presumably owing to the abundance of potassium ions throughout the bulk and lamina of the leaves.^33^ Therefore, the distribution of the potassium adduct of Azoxystrobin was considered in this study to reveal the true sub-cuticular distribution of absorbed Azoxystrobin over the sodium, ammonium or proton adducts. After 2 hours, the formulation droplet applied on leaf is completely air dried, and the Azoxystrobin had very little mobility relative to the point of application (Fig. 3). However, at 24 hours, the Azoxystrobin signal showed selective spreading towards the periphery of the leaf when compared to the leaf area towards the stem. The same trend of Azoxystrobin signals was seen after 56 hours and 1 week after formulation application, with more signal intensity being concentrated at the leaf edges near the periphery as time progressed. Similar trends were observed when the same experiments were run in triplicate and compared for the 24 h, 56 h and 1 week time points (Fig. S5†) for the *m*/*z* 442.1 signal. The visible selective movement of the Azoxystrobin ion signal towards the periphery of the leaf was further validated by the ROC analysis done on the ion images for leaf sample repeats at various time points of application (Fig. S6†). Considering different angle of growth of leaf or human errors in formulation application, there may be slight variations in intensities to either sides of the midrib of the leaf but, the general pattern of movement observed is the same on both sides of the leaf for this study. Hence, it was possible to successfully visualize the active xylem mobility and lateral lamina spreading of the formulated Azoxystrobin as described in the literature,^27^ which is critical for its protectant, fungicidal properties in young tomato leaves.

**Fig. 4:**
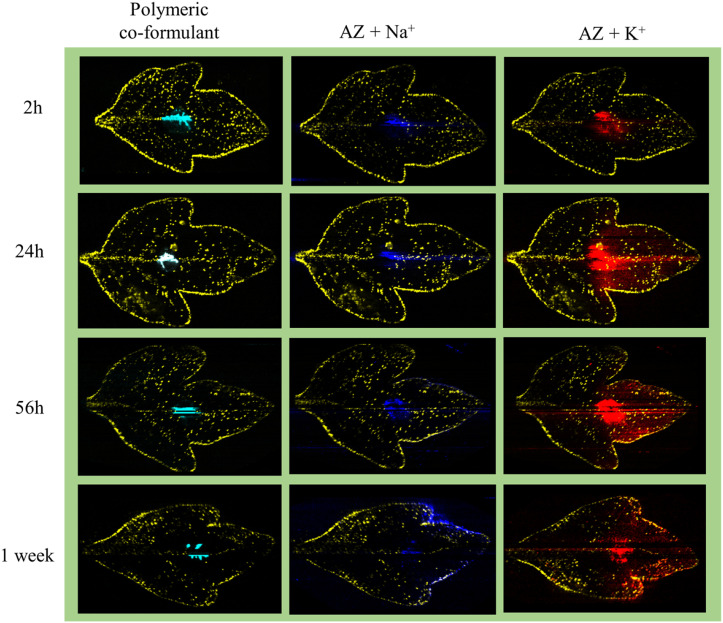
Mobility of different ion signals from formulated Azoxystrobin in tomato leaves after application. One imprint per time point was taken to show the different ion distributions. In all the ion images the yellow signal is for *m*/*z* 731.3 [acyl sugar S4:21, potassium adduct] highlighting the trichomes and the edges of the leaf. In the ion images, the red signal is for *m*/*z* 442.1 [Azoxystrobin, potassium adduct], the blue signal is for *m*/*z* 426.1 [Azoxystrobin, sodium adduct] and the cyan signal is for a polymeric co-formulant with *m*/*z* 837.5. The peak at *m*/*z* 837.5 was selected as the representative polymeric co-formulant peak as all other polymeric signals checked showed a similar distribution to it and is suspected to correspond to component no. 3 in Table S1.† The intensity maxima were set to give the best visualization of ion intensities in all ion images.

Complementary to the potassium adduct of Azoxystrobin, the ion image of the sodium adduct at *m*/*z* 426.1 shows high intensity mostly at the point of application and at the edge of the leaf in the later time points as shown in Fig. 4. Distinct from this pattern of sodium adduct distribution, all of the formulation-specific polymeric peaks identified in Fig. 2b show localization just at the point of application and no lamina spreading was observed, as shown with an example ion signal in Fig. 4. An example formulation peak at *m*/*z* 837.5 is taken here as all of the polymeric ion signals tested showed identical ion distributions in the DESI images. As these formulation-exclusive peaks appear polymeric, these could be coming from component no. 3 listed in the formulated Azoxystrobin constituents table (Table S1†) as the other component ion signals were not observed in the spectra and because component no. 3 mentioned having a polymeric composition. It was possible to clearly visualize the presence of the Azoxystrobin, even when only 2 μL of 0.25 ppm formulated Azoxystrobin solution in water was applied, air-dried, imprinted and analysed (Fig. S7, ESI Note 3†).

### Visualizing mobility of Azoxystrobin standard

In the formulated Azoxystrobin, the Azoxystrobin molecule is responsible for the fungicidal action.^34^ To check the viability of Azoxystrobin as a fungicide when applied without any adjuvants, a similar experiment to that conducted for the formulated Azoxystrobin using 2500 ppm Azoxystrobin standard in water applied as a 10 μL droplet was performed. Since the 24 h, 56 h and 1 week time points showed characteristic mobility for formulated Azoxystrobin solution in young tomato leaves, the same time points of sampling were considered for visualizing Azoxystrobin standard mobility. The results obtained were distinct for both experiments with the Azoxystrobin standard showing little to no mobility across the applied leaf as shown in Fig. 4 for the sodium as well as potassium adducts. The results indicate that for effective mobility and hence the rapid fungicidal action intended for Azoxystrobin, it being present as a formulation with adjuvants is vital.

### Adaxial or abaxial imprint?

**Fig. 5:**
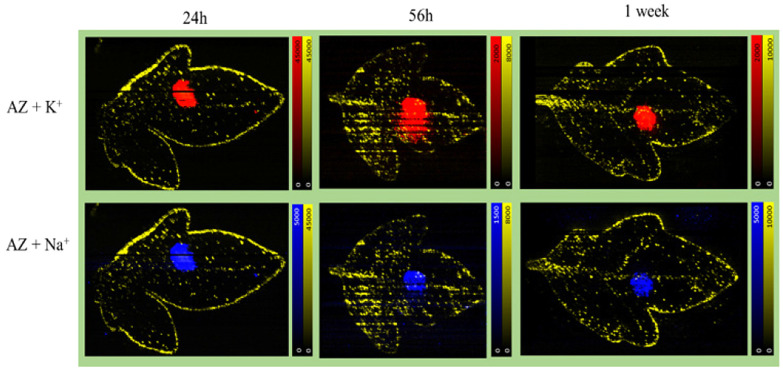
Visualizing Azoxystrobin standard distribution over time on tomato leaves. A leaf from each tomato plant in the set of plants was applied with 10 μL of 2500 ppm of Azoxystrobin standard compound on the 17^th^ day after planting of seeds. The formulation applied leaves from the plants were sampled and imprinted at 24 h, 56 h and 1 week after the time point of application. The obtained imprints were imaged within 24 hours post imprinting to obtain the above ion images. In all the ion images the yellow signal is for *m*/*z* 731.3 [acyl sugar S4:21, potassium adduct] highlighting the trichomes and the edges of the leaf along with the red ion signal for *m*/*z* 442.1 [Azoxystrobin, potassium adduct] and the blue ion signal is for *m*/*z* 426.1 [Azoxystrobin, sodium adduct]. In the ion images, the maximum arbitrary intensity value, and the minimum arbitrary intensity value (0 in all cases) are shown.

**Fig. 6:**
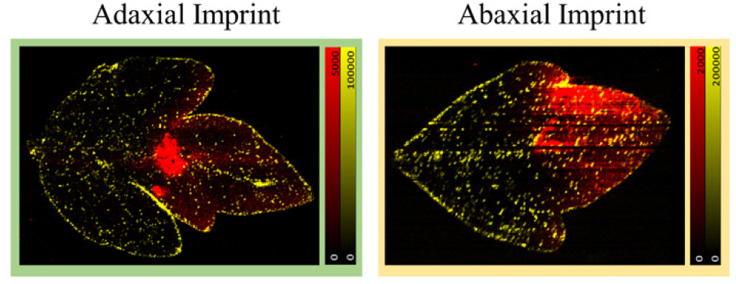
Formulated Azoxystrobin applied tomato leaf after 48 h imprinted which were adaxially and abaxially imprinted. In all the ion images the yellow signal is for *m*/*z* 731.3 [acyl sugar S4:21, potassium adduct] highlighting the trichomes and the edges of the leaf along with the red signal for *m*/*z* 442.1 [Azoxystrobin, potassium adduct]. The distribution of Azoxystrobin is quite similar for the adaxial and abaxial imprints. In the ion images, the maximum arbitrary intensity value, and the minimum arbitrary intensity value (0 in all cases) are shown.

The leaves were imprinted as mentioned with the formulation applied to the adaxial surface (Fig. S1†) against the PTFE sheet during the imprinting process for all of the fungicide mobility studies mentioned. To check, the efficiency of the imprinting process and to understand if a true representation of the 3-D distribution of the leaf into the 2-D PTFE sheet surface was present; an adaxial and abaxial imprint for samples at the same growth stage and application time were taken. Using the same growing conditions and application methods as described for the leaf mobility experiments (Fig. 3–5), one leaf each from two plants was sampled at 48 h after formulation application and imprinted. One sample was imprinted with the adaxial/formulation applied surface facing the PTFE sheet and the other was imprinted with the abaxial side of the leaf facing the PTFE sheet. The adaxial and abaxial imprints showed a similar overall distribution for *m*/*z* 442.1 (Fig. 6). The key difference between the two ion images appears to be the prominent Azoxystrobin ion signal distribution present at the point of application in the adaxial imprint. This variability can be attributed to the unabsorbed formulation applied on the leaf, which deposits on the surface and then is transferred to the PTFE sheet on direct contact (adaxial) during the imprinting process. The visual similarity in *m*/*z* 442.1 distribution in both the adaxial and abaxial imprint images is supported by the ion-correlation matrix obtained for both ion images (Fig. S8†).

### Quantitative LC-MS study

**Fig. 7:**
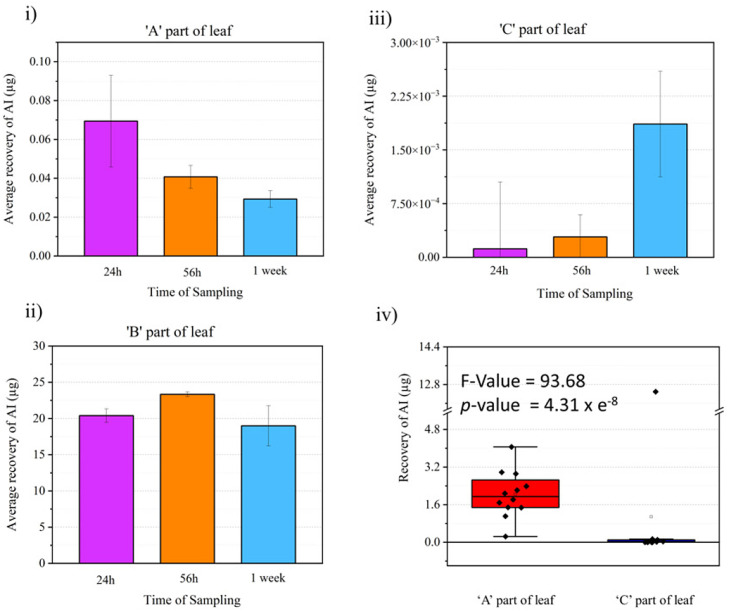
Quantitative LC-MS studies with leaf extracts. The whole leaf after formulation application was divided into three parts (Fig. 1e), A – the part of leaf towards the periphery, B – the area of application of formulation and C – the part of leaf away towards the stem of the plant as shown in the figure. The amount of Azoxystrobin recovered through the LC-MS studies in A, B and C parts of leaf at various time points are shown in (i), (ii) and (iii). (iv) Box-plot showing the total amount of Azoxystrobin (AI) recovered in A and C part of leaf within 24 h to 1 week time. A one-way ANOVA test was done on this data and the results of the same are shown as an inset in (iv).

The visual comparison of the mass spectrometry imaging data through various ion images yields meaningful results regarding information about localization and relative intensity of ion signal of interest in a sample. Since this is a proof-of-principle study on the utility of DESI MSI for visualizing active fungicide mobility in tomato leaves, a quantitative LC-MS study was done to complement the MSI results and validate it. An identical fungicide application method as the MSI study was used to prepare the samples for the LC-MS experiments but rather than imprinting and imaging, the leaf samples were divided into three as shown in Fig. 1e and put into an extraction tube for quantitative LC-MS studies. Three data points were taken per part of the leaf (A, B or C), per time point (24 h, 56 h or 1 week) for analysis. With calibration standards, the composition of Azoxystrobin [M + H]^+^ in each leaf area for all time points was elucidated as shown in Fig. 7. We observed Azoxystrobin distribution patterns comparable to previously reported literature.^35^ In agreement with the results shown in Fig. 3, the LC-MS data showed a higher abundance of Azoxystrobin in part A or towards the periphery of the leaf compared to the area of the leaf towards the stem or part C at 24 h, 56 h and 1 week after application of the formulation. The one-way ANOVA performed on the combined Azoxystrobin recovery in A and C parts of the leaf for 24 h, 56 h and 1 week time point data considering part of leaf as the variable (Fig. 7iv) gave an *F*-value of 93.68 and a *p*-value of 4.31 × 10^−8^ showing significant difference between the two groups. This result is in agreement with the intense [M + K]^+^ signal observed with DESI MSI at the point of application in all leaves in Fig. 3. We were also able to detect two previously reported metabolites^36^ of Azoxystrobin which also showed selective abundance in part A compared to part B of the leaf, much like Azoxystrobin (Fig. S9†). However, we were not able to detect the same metabolites in the DESI data acquired. A possible explanation for this may be the difference in the limit of detection between DESI MSI and LC-MS methods, which were optimized to detect Azoxystrobin.

## Conclusions

With a relatively simple sample preparation and efficient DESI MSI method, the active mobility of a commercial fungicide formulation laterally across whole tomato leaves at 4-time points extending to 1 week from formulation application on leaf was successfully visualized. The xylem mobility and lateral laminar spreading of Azoxystrobin (AI) of the formulated Azoxystrobin were observed by monitoring the abundant potassium adduct of Azoxystrobin. The sodium adduct of Azoxystrobin was observed in the edges of the leaf in the later time points of observation owing to the relative abundance of sodium ions external to the leaf compared to potassium ions.

The polymeric co-formulant signals observed in the formulation-applied leaves show very limited mobility in agreement with its role mostly in helping the AI cross the hydrophobic–hydrophilic barrier (cuticle–epidermal interface) in the leaf along with the other adjuvants in the formulation.^37^ In contrast to the formulation application study with Azoxystrobin and adjuvants, limited to no mobility was observed when the sample experiment was repeated with the Azoxystrobin standard with no adjuvants. Interestingly, the adaxial and abaxial imprinting of leaves yielded similar information with slight variations owing to formulation application artefacts hence increasing confidence in the results obtained with just adaxial imprints and its true representation of leaf chemical distributions. The results obtained in the visual comparison of MS image studies were corroborated using ROC analysis. The results we obtained for the LC-MS studies for similar samples yielded highly analogous data to that of the MSI data supporting the utility of MSI as a spatial chemical imaging tool for analysing active agrochemical mobility.

As this imprinting and MSI method was tested in very young soft tomato leaves which are comparatively difficult to handle, we believe that the same method could be translated to the visualization of active agrochemical formulation mobility of further agrochemicals or xenobiotics in general or even plant metabolites if present above the detection limit of DESI, in other fleshier plant systems, modifying pressure and imprinting period.

Adopting this DESI imprint-imaging method of plant leaves to visualize agrochemical mobility in the current agrochemical R&D workflow can have tremendous implications in saving time and cost of operation. The MSI leaf analysis eliminates the need for the preparation of radiolabelled analogous of pesticide active ingredients and facilities for the safe handling of radioactive chemicals. The major cost involved in obtaining a DESI MSI system is a DESI source which requires minimal upkeep and is replaceable with a standard ESI or APCI source for general experiments in the analytical laboratory on a mass spectrometer of choice. As it is a label-free analysis method, multiple compounds in a formulation can be visualized in one single experiment with minimal sample preparation making rich scientific agrochemical spatial screening data available early on in product development. Hence, the adaptation of DESI MSI as a method for agrochemical spatial screening can help make the agrochemical industry informed decisions early on in product development saving time and cost. This knowledge will inform the application of agrochemical products *e.g.* effective and efficient dose regimes in agricultural practice.

## Author contributions

Data investigation, curation and formal analysis: A. A. Conceptualization and methodology: N. P. L., P. J. M., E. J., G. N. J., E. P., D. K. T. and A. A. Funding acquisition: N. P. L., P. J. M., G. N. J. and D. K. T. Writing of original draft: A. A. Supervision and writing – review & editing: N. P. L., P. J. M., E. J., G. N. J., E. P. and D. K. T.

## Data availability

Data for this article, including .imzML and .mzML files are available at Zenodo at https://doi.org/10.5281/zenodo.13890490 and https://doi.org/10.5281/zenodo.13899697.

## Conflicts of interest

There are no conflicts to declare.

## Supplementary Material

AN-149-D4AN01309C-s001
