# Supplementary material for: Visualizing active fungicide formulation mobility in tomato leaves with desorption electrospray ionisation mass spectrometry imaging
Source: Analyst. 2024 Nov 18;149(24):5904–13. doi: 10.1039/d4an01309c (PMC11583139; doi:10.1039/d4an01309c)
Supplement: AN-149-D4AN01309C-s001 [file AN-149-D4AN01309C-s001.pdf]

Supplementary Information (SI) for Analyst.
This journal is © The Royal Society of Chemistry 2024

Supporting Information for

Visualizing active fungicide formulation mobility in tomato leaves with Desorption Electrospray Ionisation Mass Spectrometry Imaging

Akhila Ajith[1], Emrys Jones[2], Emily Prince[3], Drupad K. Trivedi,[1] Giles N. Johnson[4], Phillip J. Milnes[3], Nicholas P. Lockyer[1*]

[1]Department of Chemistry, University of Manchester, Manchester, U.K, [2] Waters Corporation, Altrincham Road, Wilmslow, UK, [3]Syngenta, Jealott's Hill International Research Centre, Bracknell, U.K, [4]Department of Earth and Environmental Sciences, University of Manchester, Manchester, U.K.

*Nicholas P. Lockyer.

Email: nick.lockyer@manchester.ac.uk

This PDF file includes:

Supplementary note 1 to 3

Table S1

Figures S1 to S9

SI References

Supplementary note 1:

DESI Solvent testing

Desorption Electrospray Ionisation (DESI) is an ambient ionization method that uses pneumatically assisted charged microdroplets as the ionization source. In this process, the composition of the solvent system along with the other geometrical parameters of the DESI setup is of paramount importance for the sensitivity of a chemical compound towards DESI MSI. To identify the ideal solvent for use in the DESI imaging experiments targeting Azoxystrobin in AMISTAR formulation, a comparison of the sensitivity was done using 80:20 (*v/v)* Methanol/Water and 80:20 (*v/v)* Acetonitrile/Water with 0.1 % and 1% formic acid additions (Figure S3). 5 μL droplets of AMISTAR formulation containing 500 ppm Azoxystrobin in water was drop-casted onto microscope slides and were allowed to air-dry. A line scan was done across the droplet to obtain one chromatogram/data file and an analysed droplet area was not reanalysed with care taken to do subsequent line scans done moving further away from the previous analysis. Three-to-four-line scans were done per droplet and 6 data files were collected per solvent system. The comparison of sensitivity was done using the absolute intensity of Azoxystrobin ion signals obtained from the averaged spectrum obtained with the line scans for a retention time window of 0.2 in the MassLynx software by Waters Corp. The plotting of the graph was done using Origin Pro software (OriginLab Corporation).

Supplementary note 2:

Identifying formulation-specific peaks

To identify the formulation-specific peaks, 5 μL of 2500 ppm Azoxystrobin containing AMISTAR formulation in water and 5 μL of Azoxystrobin standard solution in water were drop-casted onto standard microscope slides and were allowed to air-dry. A line scan was done across the droplet to obtain one data point and an analysed droplet area was not reanalysed. One line scan was taken from the middle of three droplets each for both AMISTAR formulation solution and Azoxystrobin giving three data points per sample. An average mass spectrum was obtained by combining the spectra in a retention time window of width 0.2 from the middle of the droplet in the MassLynx software. Using Origin Pro software, the three mass spectra each for AMISTAR and Azoxystrobin standard were averaged using the curve math function to obtain a representative spectrum for both. Normalizing the average spectra from 0 to 1 yielded Figure 2a. To identify the formulation-exclusive peaks, the normalized average spectrum of the Azoxystrobin standard was multiplied by 100 and

subtracted from the average normalized spectrum of AMISTAR formulation. After switching the negative values for zero, Figure 2b was obtained which indicates that most of the formulation-exclusive peaks might be polymeric and may correspond to component no. 3 of the Azoxystrobin formulation composition as mentioned in Table S1.

Supplementary note 3:

Testing varying concentrations of formulation application on tomato leaves

To test the limit of detection of the DESI Imaging experiments done in this study, an imaging experiment was done by applying 2μL of 2500 ppm, 250 ppm, 25 ppm, 2.5 ppm, 0.25 ppm and 0 ppm of Azoxystrobin containing AMISTAR formulation solution in water to the same 17-day old tomato leaf side-by-side on the plant (Figure S7). After one hour of formulation solution application when the droplets had air-dried, the leaf was extracted from the plant and was imprinted and imaged with 2000 psi pressure for 1 minute. The same imaging conditions as described in section 2.5 were used. We were able to visualize the presence of AI even in the 0.25 ppm droplets applied area in the leaf. In the experiments when translated for Azoxystrobin standard similar results were obtained although there are variations in the image quality owing to the artefacts of applying of formulation and the variations in leaf morphology as they were done on two different plant leaves.

Table S1: Contents of Azoxystrobin formulation as listed in safety data sheet of AMISTAR

Component No.	Chemical name	CAS-No.<br>EC-No.<br>Index-No.<br>Registration No.	Classification	Concentration (%w/w)
1	Azoxystrobin (ISO)	131860-33-8<br><br>607-256-00-8	Acute Tox. 3; H331<br>Aquatic Acute 1; H400<br>Aquatic Chronic 1; H410<br><br>M-Factor (Acute aquatic toxicity): 10<br>M-Factor (Chronic aquatic toxicity): 10	>=20 - <25
2	C16-18 alcohols, ethoxylated	68439-49-6<br>500-212-8	Acute Tox. 4; H302<br>Eye Dam. 1; H318	>= 10 - <20
3	Residues (petroleum), catalytic reformer fractionator, sulfonated, polymers with formaldehyde, sodium salts	68425-94-5	Eye Irrit. 2; H319	>= 1-<10
4	1,2-benzisothiazol-3(2H)-one	2634-33-5<br>220-120-9<br>613-088-00-6<br>01-2120761540-60	Acute Tox. 4; H302<br>Skin Irrit. 2; H315<br>Eye Dam. 1; H318<br>Skin Sens. 1; H317<br>Aquatic Acute 1; H400<br>Aquatic Chronic 2; H411<br><br>M-Factor (Acute aquatic toxicity): 1<br><br>Specific concentration limit	>=0.025 - <0.05

			Skin Sens. 1; H317<br>>= 0,05%	

Abbreviations; H302 : Harmful if swallowed. H315 : Causes skin irritation. H317 : May cause an allergic skin reaction. H318 : Causes serious eye damage. H319 : Causes serious eye irritation. H331 : Toxic if inhaled. H400 : Very toxic to aquatic life. H410 : Very toxic to aquatic life with long lasting

Acute Tox. : Acute toxicity Aquatic Acute : Acute aquatic toxicity Aquatic Chronic : Chronic aquatic toxicity Eye Dam. : Serious eye damage Eye Irrit. : Eye irritation Skin Irrit. : Skin irritation Skin Sens. : Skin sensitisation.

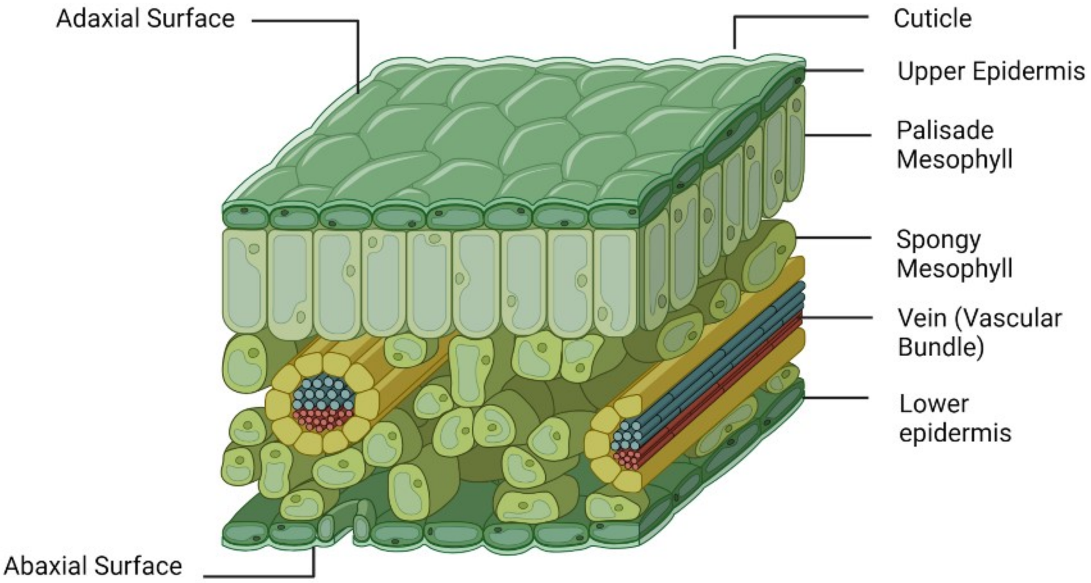

Figure S1. A graphical representation of the various parts of a plant leaf. This image was created using BioRender.com

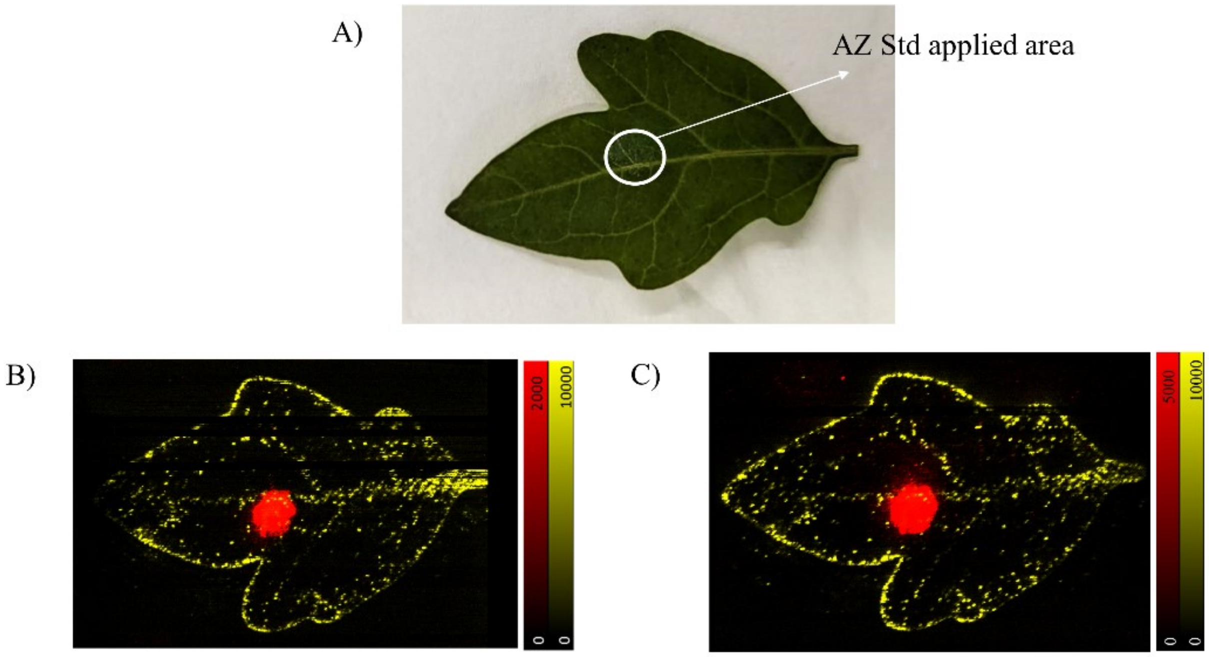

Figure S2. Reimaging of imprints. A 17-day-old tomato leaf **(A)** was applied with 10 µL 2500 ppm Azoxystrobin standard (AZ Std) was imprinted, imaged **(B)** and reimaged **(C)** after 1 week of solution application. Images B). C) were obtained after the imaging of the same imprint as B) after 6 days. In the ion images the yellow distribution corresponds to *m/z* 731.3 [Acyl sugar S4:21, potassium adduct] highlighting the trichomes and the edges of the leaf and the red ion signal is for *m/z* 442.1 [Azoxystrobin, potassium adduct]. We see very similar ion distributions obtained during the imaging and re-imaging for the same imprint at least for this mentioned imprint-image and re-imaged imprint.

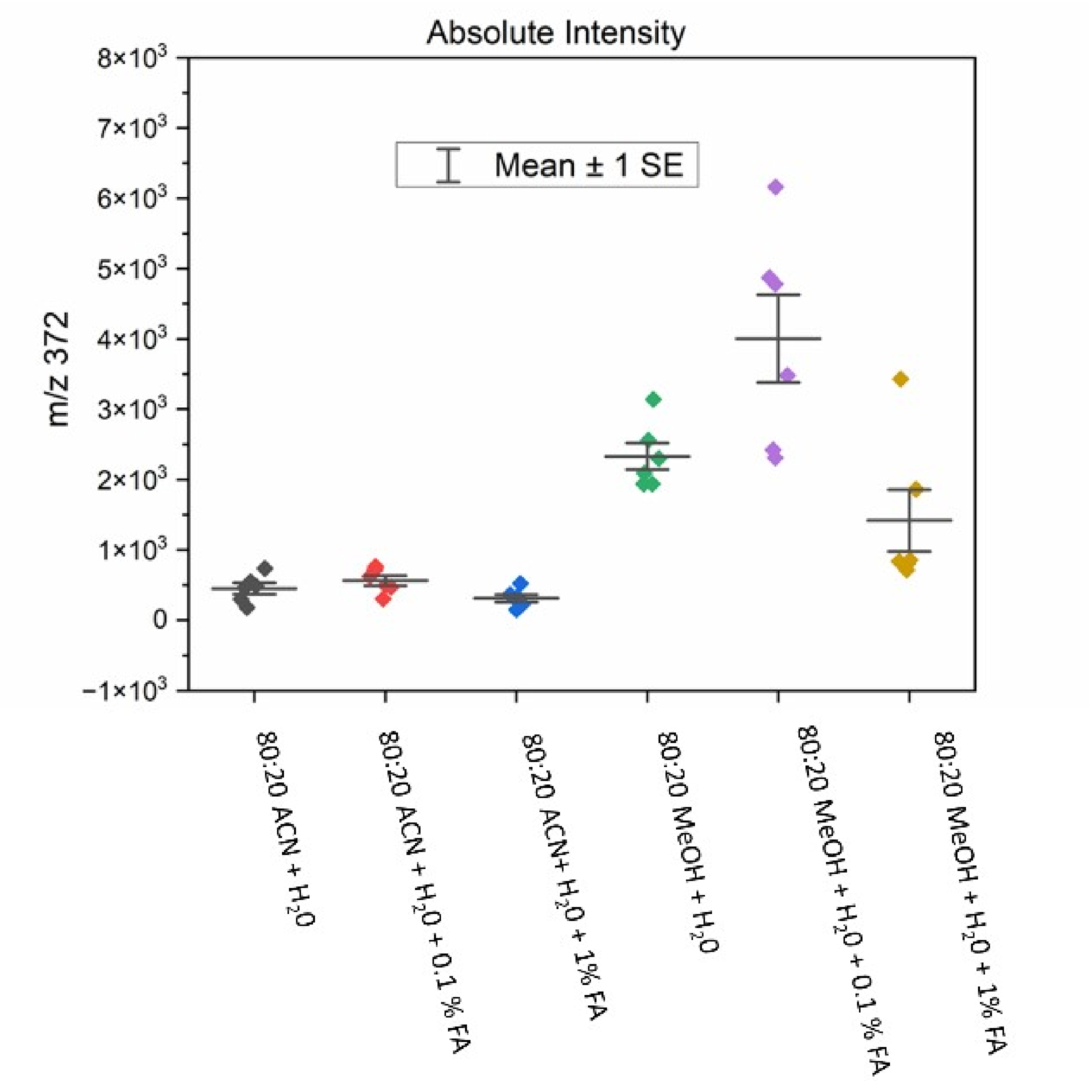

Figure S3. Testing the sensitivity of different solvent systems for Azoxystrobin signal using a representative peak, *m/z* 372.1. All azoxystrobin signals showed similar trends. For the solvent testing; 80:20 (*v/v)* Methanol/Water and 80:20 (*v/v)* Acetonitrile/Water with 0.1 % and 1% formic acid additions were taken. For all the tested ion signals of azoxystrobin (80:20 (*v/v)* Methanol/Water with 0.1% formic acid addition showed the highest relative sensitivity.

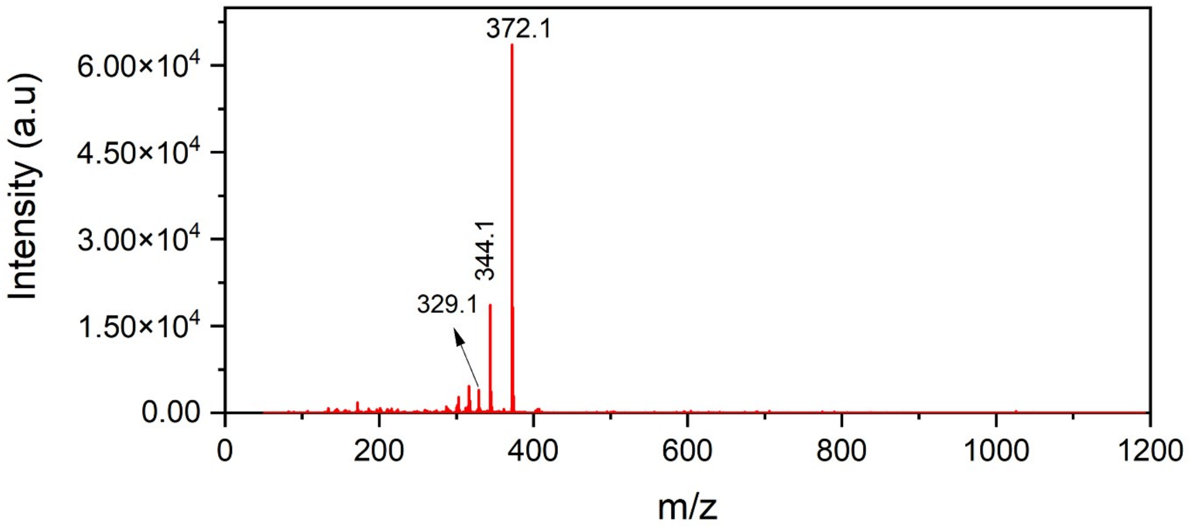

Figure S4. MS/MS of Azoxystrobin, Proton adduct (*m/z* 404.1) obtained from a leaf imprint on a PTFE sheet with relative collision energy of 30 CE with DESI ionization source on a Synapt G2-Si.

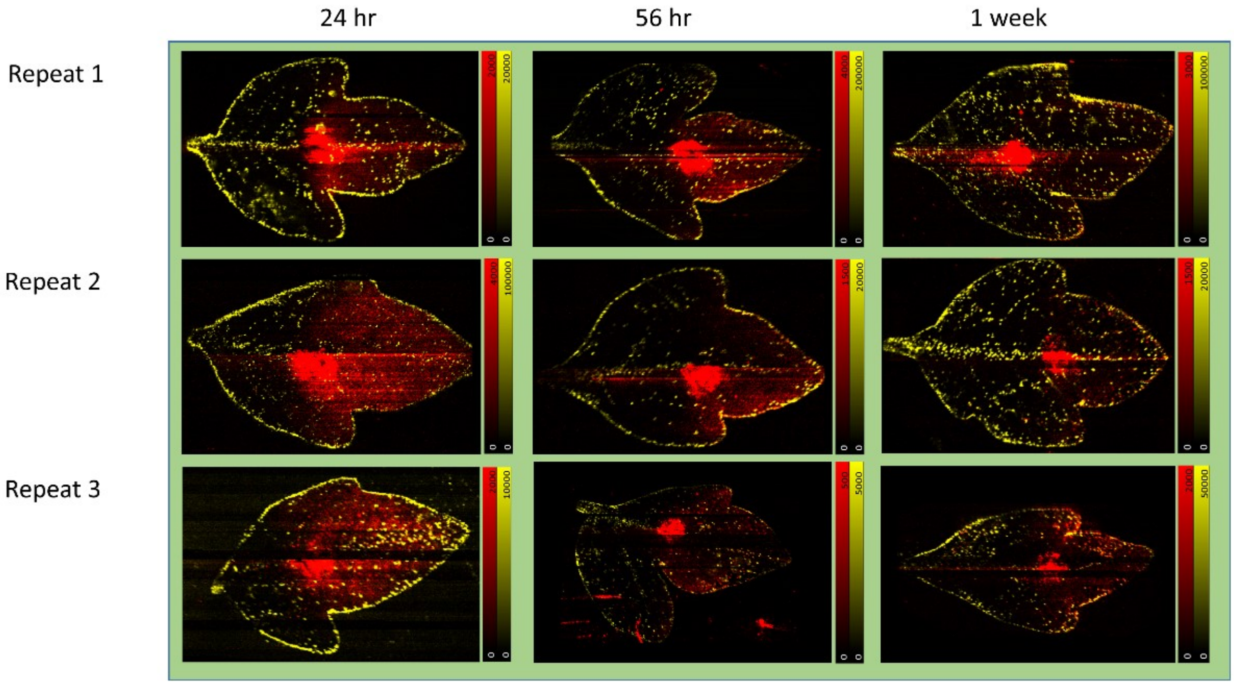

Figure S5. Repeatability of measurements. Young tomato leaves were applied with 10 μL of Azoxystrobin formulation (2500 ppm of Azoxystrobin in the formulation) on the 17th day after planting the seeds. The leaves were samples at 2h, 24h, 56h and a week after fungicide formulation application. To test whether this method is viable for agrochemical testing, we imaged 3 repeats for each of the 24h, 56h and a week time points. In the ion images the yellow ion signal corresponds to *m/z* 731.3 [Acyl sugar S4:21, potassium adduct] highlighting the trichomes and the edges of the leaf and the red ion signal is for *m/z* 442.1 [Azoxystrobin, potassium adduct]. In all the repeats of a specific time point, we see a similar pattern of spreading in the leaves. In the ion images, the maximum arbitrary intensity value, and the minimum arbitrary intensity value (0 in all cases) are shown.

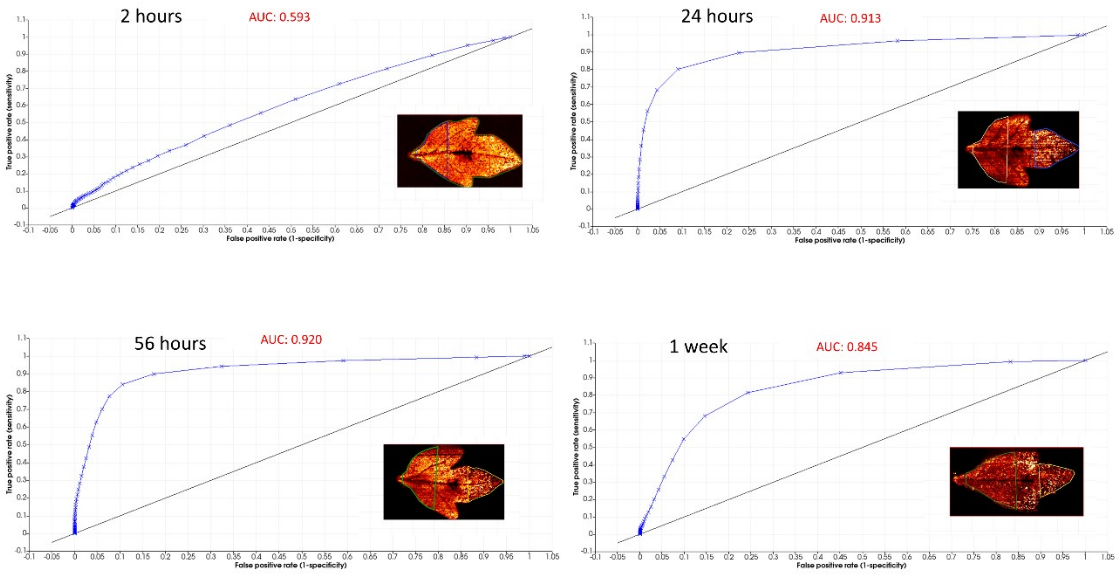

Figure S6. Receiver Operating Characteristics analysis was done to check the variability in the ion intensity distribution of *m/z* 442.1 [Azoxystrobin, potassium adduct] by comparing the area towards the periphery of the leaf and the area towards the stem of the leaf concerning the point of application (highlighted in the inset image of the respective leaves at *m/z* 1034.6). ROC curves are plotted using sensitivity and specificity estimated for a trivial threshold classifier for *m/z* 442.1 by random selection of 200 single pixel spectra from each region after TIC normalization. The calculations are shown for one sample representative of a time point and similar results were obtained for all the repeats of a time point. The Area Under the Curve values calculated for ROC if near 0.5 show no variability in intensity distribution between the selected areas for the particular ion and if the value is closer to 0 or 1 indicates maximum variability. It could be seen that as time progresses after formulation application, AUC values indicate more variability *m/z* 442.1 distribution for the periphery of the leaf and the area of the leaf towards the stem of the plant. The ROC calculations were done using the Bruker SciLS lab software.

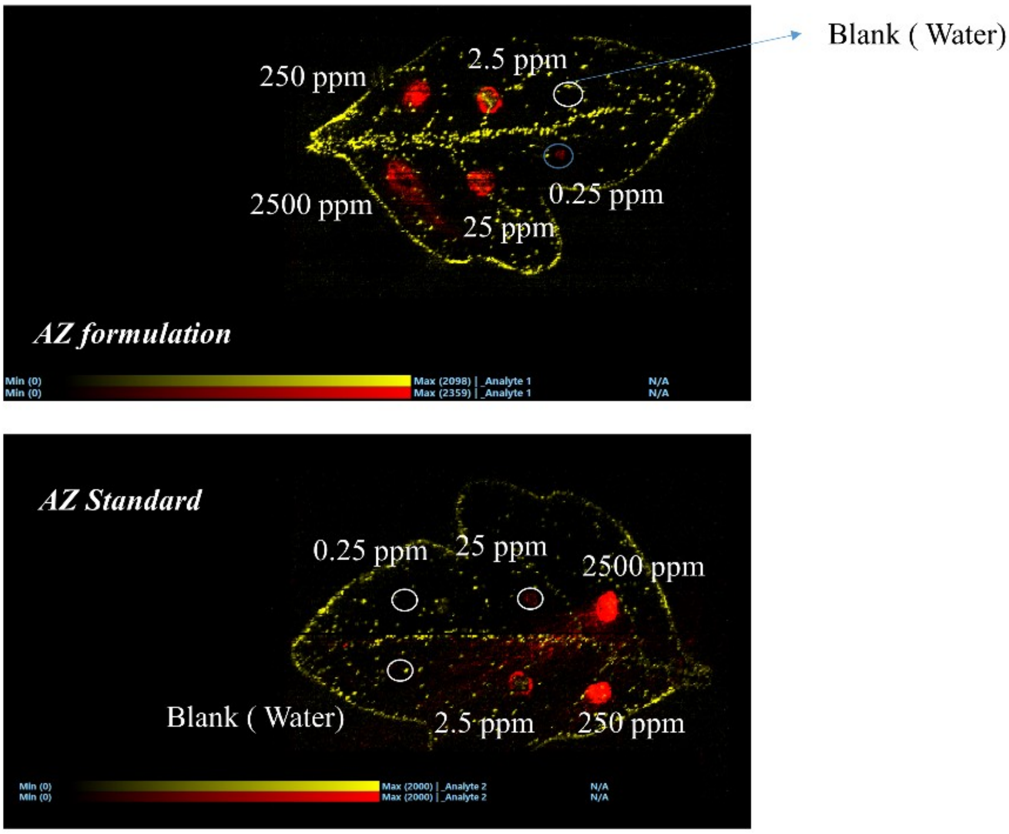

Figure S7. Varying concentrations of AMISTAR formulation and Azoxystrobin standard on 17-day-old tomato leaves. The two leaves, one each for formulation and standard were applied with 2 µL of formulation/standard and were air-dried for 1 hour on the plant before imprinting and imaging with the same experimental conditions as the mobility study in Figure 3. Even 0.25 ppm AZ was comfortably visualized in DESI images.

Adaxial imprint

		426.1	442. 1	837.5	731.3	1034.6
AZ + Na+	426.1	1	0.547	0.243	0.000324	9.48E-05
AZ + K+	442. 1	0.547	1	0.329	0.000332	0.0032
Formulation peak	837.5	0.243	0.329	1	5.23E-05	0.000569
Acyl sugar S4:21 + K+	731.3	0.000324	0.000332	5.23E-05	1	0.00526
Tomatine + H+	1034.6	9.48E-05	0.0032	0.000569	0.00526	1

Abaxial Imprint

		426.1	442. 1	837.5	731.3	1034.6
AZ + Na+	426.1	1	0.789	0.0316	5.99E-05	1.16E-05
AZ + K+	442. 1	0.789	1	0.0427	0.000925	0.00104
Formulation peak	837.5	0.0316	0.0427	1	0.000188	0.000252
Acyl sugar S4:21 + K+	731.3	5.99E-05	0.000925	0.000188	1	0.0541
Tomatine + H+	1034.6	1.16E-05	0.00104	0.000252	0.0541	1

Figure S8. Ion correlation matrices for ion images in Figure 6. The ion correlation matrix values indicate the degree of intensity similarity in distribution between the selected ions in the different pixels in the sample (mass interval ±0.02Da). The ion correlation plot was calculated by exporting the Waters DESI imaging data to SciLS lab software by Bruker. The pair-wise correlation values are obtained by plotting the intensities of the ions of interest against each other in the various pixels of the sample and these pair-wise correlations of the ions of interest are used to generate the final matrix by clustering followed by sorting of each row of the matrix using hierarchical clustering. The correlation is indicated by a linear scale from 0 to 1 where a value of 0 means no correlation and a value of 1 means perfect correlation. Even if imprinted adaxially or abaxially, the correlation between Azoxystrobin(AZ) ions and background ions (Acyl Sugar and Tomatine) appears similar. The correlation between AZ ions and the formulation additive peak (Formulation peak) as expected only exists in the adaxial imprint as the additives are expected to remain mostly on the surface of the adaxial waxes of the leaf as they do not spread into the leaf and just facilitates the movement of AZ into the bulk of the leaf. Hence, this indicates that the chemical information is not drastically different between the adaxial and abaxial imprints and we might be getting a true 2-D representation of the surface distribution of ion signals of a 3-D leaf when imprinted.

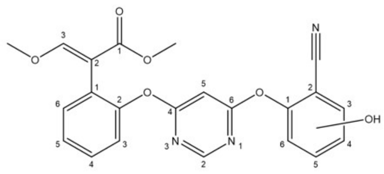

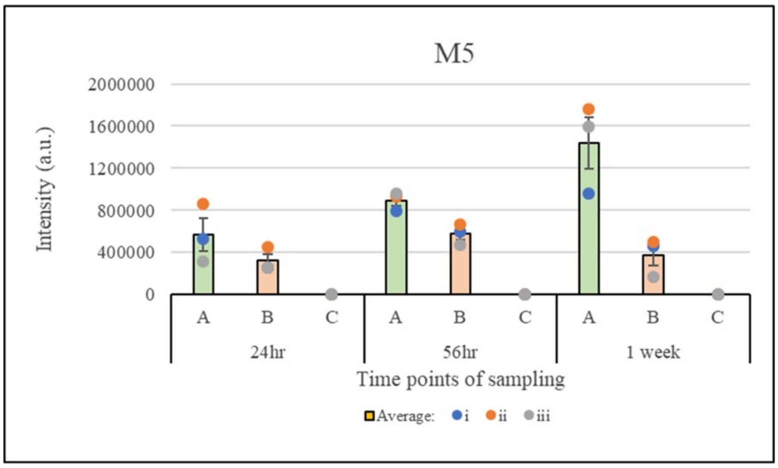

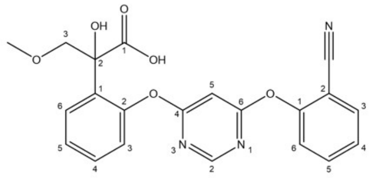

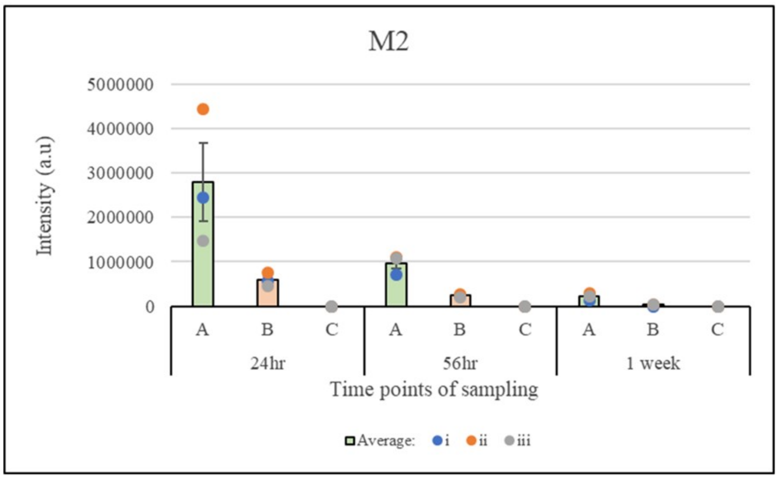

Figure S9. Metabolites detected in LC-MS study. In the quantitative LC-MS experiments (Figure 7) performed to observe the amount of Azoxystrobin recovered after formulation application, we also observed the presence of two metabolites M2 and M5 already reported in literature.[1] The metabolites were mainly present in the A parts (Figure 1e) of the leaf decreasing in relative intensities as time points progressed to 1 week. M2 appeared to be decreasing from 24h to 56h to 1 week of application in the A and B parts of leaf and contrast to M2, M5 appeared to be increasing over time till 1 week with no M2 and M5 observed for the 2h time point. We attempted to track M2 and M5 in the DESI images, but these could not be observed in the top 1500 peaks observed for the DESI imaging data. In the plots, i, ii and iii represent the repeats for each part of the leaf at various time points.

References:

(1) Gautam, M.; Etzerodt, T.; Fomsgaard, I. S. Quantification of azoxystrobin and identification of two novel metabolites in lettuce via liquid chromatography–quadrupole-linear ion trap (QTRAP) mass spectrometry. *International journal of environmental analytical chemistry* **2017**, *97* (5), 419-430.
